# Revision of the New World genus *Enderleiniella* Becker, 1912 (Diptera, Chloropidae)

**DOI:** 10.3897/zookeys.884.36154

**Published:** 2019-10-30

**Authors:** Julia J. Mlynarek

**Affiliations:** 1 Agriculture and Agri-Food Canada, Harrow Research and Development Centre, Harrow, ON, N0R 1G0, Canada Agriculture and Agri-Food Canada Harrow Canada

**Keywords:** Grass fly, frit fly, Neotropic, Nearctic, Central America, taxonomy

## Abstract

The genus *Enderleiniella* Becker, 1912 is revised. The genus is distinguished on the basis of a somewhat flattened head with the inner vertical setae located anteromedially to the outer vertical setae, three lightly incised lines on the scutum, trapezoidal or rectangular scutellum with marginal setae borne on tubercles, reduced alula and anal angle of the wing, and the structure of the male genitalia. The genus contains eleven species in the northern Neotropical and southern Nearctic Regions: *E.caerulea***sp. nov.** (type locality: Blue Creek, Belize); *E.cryptica***sp. nov.** (type locality: 24 km W Piedras Blancas, Costa Rica); *E.flavida***sp. nov.** (type locality: Emerald Pool, Dominica); *E.longiventris* (Enderlein, 1911) (type species; type locality: Costa Rica); *E.maculata***sp. nov.** (type locality: Xilitla, San Luis Potosi, Mexico); *E.marshalli***sp. nov.** (type locality: Guanacaste, Costa Rica); *E.maya***sp. nov.** (type locality: Las Escobas, Guatemala); *E.punctata***sp. nov.** (type locality: Potrerillo, Bolivia); *E.tripunctata* (Becker, 1916) (type locality: San Mateo, Costa Rica); *E.tumescens***sp. nov.** (type locality: San Esteban, Venezuela); and *E.wheeleri***sp. nov.** (type locality: Turrialba, Costa Rica).

## Introduction

The genus *Enderleiniella* was proposed by Becker (1912) for the single species *Tricimbalongiventris* Enderlein, 1911. Subsequently, Becker (1916) proposed a second genus, *Anoscinella* Becker, 1916, for the new species *Anoscinellatripunctata* Becker, 1916. Duda (1930) considered the two species congeneric and synonymised *Anoscinella* under *Enderleiniella*. That synonymy was accepted by subsequent authors (e.g., Sabrosky and Paganelli 1984) and there has been no published taxonomic research on the genus since. As part of an inventory of Costa Rican Chloropidae and the chloropid chapter in the Central American Manual of Diptera (Wheeler 2010), several undescribed species of *Enderleiniella* were identified in the northern Neotropical and extreme southern Nearctic regions. The validity of the genus has recently been questioned because certain characters of *Enderleiniella* seem to fit into the definition of *Tricimba* Lioy, 1864 (M von Tschinrhaus and JW Ismay pers. comm.).

The purpose of this paper is to revise the genus *Enderleiniella*, provide descriptions of those new species, present a morphological key to species, and discuss the validity of *Enderleiniella* as a genus.

## Materials and methods

Specimens studied are housed in the Canadian National Collection of Insects, Ottawa, Ontario, Canada (**CNC**); University of Guelph Insect Collection, Guelph, Ontario, Canada (**DEBU**); Instituto Nacional de Biodiversidad, Santo Domingo de Heredia, Costa Rica (**INBio**); Lyman Entomological Museum, McGill University, Sainte-Anne-de-Bellevue, Quebec, Canada (**LEM**); National Museum of Natural History, Smithsonian Institution, Washington, DC, USA (**USNM**).

For examination of genitalia, the abdomen was removed (older air-dried specimens were relaxed in a humidity chamber prior to dissection) and cleared in 85% lactic acid heated in a microwave oven for two periods of 10 seconds, separated by a one-minute cooling period. The cleared abdomen was then placed in glycerine for further dissection and examination. The dissected abdomen was stored in glycerine in a plastic microvial pinned beneath the source specimen. Morphological observations were done on a Leica M165C microscope, genitalic observations were done on a Leica DM6C microscope. The specimens were photographed with a Leica DFC 450 camera mounted on the microscope. Morphological terminology follows Cumming and Wood (2009). I sent specimens for sequencing of the insect barcode fragment of the Cytochrome c Oxidase one (CO1) to the Centre of Biodiversity Genomics, Biodiversity Institute of Ontario. Because most specimens are old, only eight individuals were successfully sequenced with the complete 658 bp fragment representing four species (GenBank: MK919190, MK919191, MK919192, MK919194, MK919195, and MK919196). To determine whether *Enderleiniella* should be synonymised with *Tricimba*, I chose seven species as outgroups in the subfamily Oscinellinae. These seven species were chosen based on the availability from the public records on BOLD and that they represent different groups within the subfamily Oscinellinae. To determine whether *Enderleiniella* should be synonymised with *Tricimba*, I chose the type species of *Tricimba* and two others from other biogeographical realms: *Tricimbalinealla* (BOLD:AAH4184), *Tricimbatrisulcata* (GenBank: JF867146, BOLD:AAN5667), and a specimen of *Tricimba* sp. (from the Neotropics; GenBank: MK919193). Sequences of *Oscinellafrit* (GenBank: OPPFO330, BOLD:AAN5659), *Aphanotrigonumscabrum* (GenBank: JF874104, BOLD:AAQ0868), *Eribolusnana* (GenBank: JF873115, BOLD:AAH4175), and *Elachipteranigriceps* (BOLD:AAP5169) were also analysed as representatives of other tribes in the subfamily Oscinellinae.

The 658 bp cytochrome oxidase one barcode sequences were aligned in MUSCLE (Edgar 2004). A Maximum Likelihood (ML) tree using GTR+G+I evolution model (the best fit for the sequences; AICc = 3788.63; BIC = 4053.86) to determine whether there is molecular support to validity of *Enderleiniella* as a genus. Bootstrap value branch support was determined by replicating the analyses 1000 times (Felsenstein 1985). Evolutionary analyses and ML tree were conducted in MEGA 7 (Kumar et al. 2016).

## Taxonomy

### Enderleiniella

Taxon classificationAnimaliaDipteraChloropidae

Becker, 1912

C9C05D9D-73FC-5369-87E8-091315C6F4CB

Enderleiniella Becker, 1912: 192. Type species: Tricimbalongiventris Enderlein (original description).
Anoscinella Becker, 1916: 448. Type species: Anoscinellatripunctata Becker (monotypy); Duda 1930: 70 (synonymy).

#### Diagnosis.

Small to medium Oscinellinae with head as wide as or slightly wider than scutum in dorsal view, occiput posteriorly convex in dorsal view, eye usually hairy, gena thin always parallel with ventral portion of eye, scutum elongate, with three parallel lines of shallow incised punctures, wing long and slender, broadest in distal half, with reduced alula and anal angle, legs long and slender, abdomen long and narrow, male genitalia with sternite 6 present, in some species epandrium enlarged and cercus elongate.

#### Description.

**Figures 1–4.**
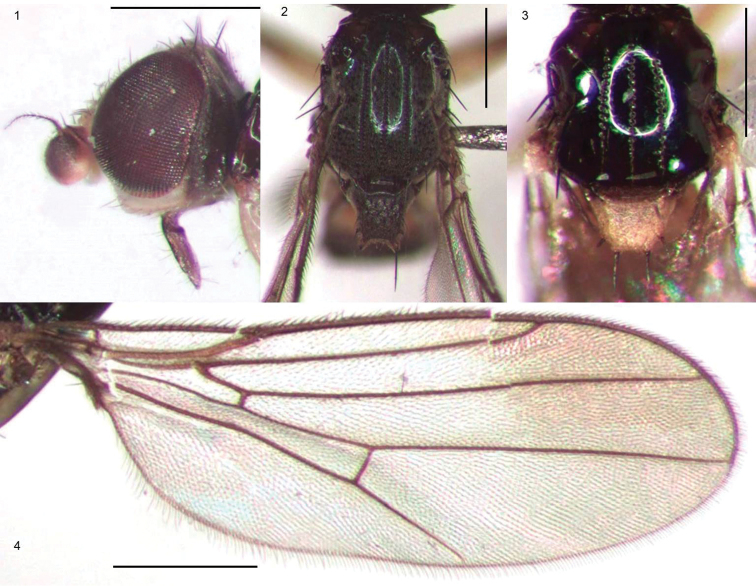
**1***Enderleiniellalongiventris*, head lateral view. **2***Enderleiniellalongiventris*, scutum and scutellum **3***Enderleiniellaflavida*, scutum and scutellum **4***Enderleiniellamaculata*, wing. Scale bars: 0.5 mm.

(Figs 1–4): Chloropidae, Oscinellinae. ***Head.*** Frontal triangle glossy or microtomentose, occiput convex; frons with many interfrontal setulae; cephalic setae short, 6–12 reclinate fronto-orbitals with most dorsal seta proclinate; ocellars and postocellars erect, convergent, vertical setae stronger than other cephalic setae, inner vertical setae as long as outer vertical setae, in line with posterior ocelli; ocelli in most species large; eye large, oval, usually densely haired; postgena clearly visible; gena linear, densely pruinose, very pale except for brown ventral margin; vibrissal angle not projecting, vibrissa present but small; face flat, narrow, pruinose, very pale, facial carina short and small; antenna with scape and pedicel short, first flagellomere large, subquadrate; arista sparsely pubescent, aristal setulae usually longer than width of arista at base; proboscis variable from small to geniculate, but never long, palpus short. ***Scutum.*** Pronotum elongate and visible in dorsal view, sulcus between postpronotum and scutum deep and well defined; scutum usually glossy, rarely pruinose, with three parallel lines of finely incised punctures (treated as grooves by Ismay (1993)), less distinct in some species, with one anterior and either one (*Enderleiniellaflavida*) or two posterior notopleural setae, one longer outer postalar bristle and one weak inner postalar setae, one postsutural dorsocentral bristle, all dark; postpronotal seta and other scutal setae weak; scutal setulae short, weak, evenly arranged in three distinct rows; in certain species scattered setulae present between and outside the three distinct rows; scutellum flattened, trapezoidal or rectangular dorsally, with two small marginal projections, bearing one strong bristle, lateral marginal scutellar setae present; Thoracic pleurites bare. ***Wing.*** Hyaline (a darkened spot in *Enderleiniellamaculata*), long and slender, broadest in distal half, alula and anal angle reduced, cell c normal, second costal sector longer than third; cell r_1_ normal and long, R_2+3_ and R_4+5_ divergent at base, cell br narrow, crossvein r-m near middle of cell dm, crossvein dm-cu fused with M4 in a right angle. ***Legs.*** Slender without outstanding setae or spurs; femoral organ present as a row or small patch of three or four sensillae, tibial organ long oval, velvety, with single longitudinal row of setulae along midline. ***Abdomen.*** Narrow, cylindrical; syntergites 1+2 elongate; tergites broad; sternites small and narrow; spiracles 3–5 in abdominal membrane ventral to lateral margins of tergites;

#### Male specific characters.

Sternite 6 present; dorsal pregenital sclerite symmetrical, left and right spiracles 6 and 7 within sclerite close to lateral margin; epandrium variable from small and simple to enlarged and inflated. Surstylus simple, straight, elongate; hypandrium open posteriorly; pregonite and postgonite simple, round and elongate; cercus variable from simple and elongate to multiple lobed; subepandrial sclerite usually small.

#### Female specific characters.

Terminalia unmodified, typical of Oscinellinae.

#### Comments.

The type species, *Enderleiniellalongiventris*, is atypical in having a densely pruinose scutum and a long, rectangular scutellum.

##### Key to described species of *Enderleiniella*

**Table:** 

1	Scutellum pale yellow (Fig. 16), contrasting strongly with dark scutum; occiput with single strong bristle behind eye; one anterior and one posterior notopleural bristle	***E.flavida* sp. nov.**
–	Scutellum brown or grey, not contrasting strongly with scutum; occiput without outstanding bristle behind eye; one anterior and two posterior notopleural setae	**2**
2	Scutum and frontal triangle densely pollinose (dull) (Figs 26, 33)	**3**
–	Scutum and frontal triangle glossy or sparsely pollinose (Figs 21, 53, 59)	**4**
3	Wing hyaline; male epandrium expanded, broader and tall than pre-epandrium; large species, body length 2.7–3.6mm	***E.longiventris* (Enderlein)**
–	Wing with a dark apical spot (Fig. 33); male epandrium smaller, not excessively higher than pre-epandrium; smaller species, body length 2.4–2.9mm	***E.maculata* sp. nov.**
4	Occiput with dorsolateral pubescent swelling just behind outer vertical bristle (in males because females currently unknown); mouthparts not geniculate (the only species from Venezuela)	***E.tumescens* sp. nov.**
–	Occiput not modified, swelling absent; mouthparts geniculate or not (species not known from Venezuela)	**5**
5	Scutum 1.2 times as long as wide; Abdominal tergite 3 with setae arising from enlarged punctate sockets (in males because females currently unknown)	***E.punctata* sp. nov.**
–	Scutum at least 1.5 times as long as wide. Abdominal tergite 3 unmodified	**6**
6	Scutellum rectangular; apical scutellar tubercles long at least 1/5 the length of the scutellum	***E.maya* sp. nov.**
–	Scutellum trapezoidal; apical scutellar tubercles short or long at most 1/8 the length of scutellum	**7**
7	Scutum sparsely pollinose	***E.marshalli* sp. nov.**
–	Scutum polished	**8**
8	Mouthparts geniculate	**9**
–	Mouthparts not geniculate or elongate	**10**
9	Scutellum 0.8 times as wide as long, with distinct tubercles, Male epandrium large, surstylus parallel sided with round tip	***E.cryptica* sp. nov.**
–	Scutellum 0.6 times as long as wide, with very small tubercles; Male genitalia small, surstylus triangular with pointed tip	***E.wheeleri* sp. nov.**
10	Hairs on scutum in well-defined rows between punctate dorsocentral rows (grooves). In male, epandrium very wide compared to high, cerci in lateral view directed posteroventrally	***E.caerulea* sp. nov.**
–	Hairs on scutum scattered between punctate rows. In male, epandrium square, cerci in lateral view directed ventrally	***E.tripunctata* (Becker)**

### Enderleiniella caerulea sp. nov.

Taxon classificationAnimaliaDipteraChloropidae

03E66A7A-A66E-5209-872D-223791BB46BA

http://zoobank.org/0998D876-29F8-4195-AA88-3A04701C6EA5

Figs 5–9

#### Diagnosis.

Medium Oscinellinae with a shiny frontal triangle and thorax. Hairs on scutum placed in well-defined rows between punctate dorsocentral rows (grooves). Scutellum small and trapezoidal with small tubercles. Male postabdomen large and bulbous.

#### Description.

Total length 2.3–2.7 mm. Overall colour black. ***Head.*** Frontal triangle black, shiny, microtomentose, 0.6–0.7 times length of frons; ocellar tubercle black, shiny, microtomentose; frons brown to black, paler medially; cephalic setae dark, 10–12 fronto-orbital setae well-developed, interfrontal setulae inside margin of frontal triangle and fronto-orbital setulae sparse, eight or nine post-ocellar setulae small; gena yellow, microtomentose, 0.06–0.08 times eye height; face yellow; scape and pedicel yellow, first flagellomere yellow basally and ventrally, black dorsally and distally, first flagellomere round, arista brown, thin at base, pubescence sparse and short; palpus yellow in male, brown in female, proboscis and clypeus brown. ***Scutum.*** Black, shiny, acrostichal and dorsocentral setae in three punctuate rows, scutum longer than wide; scutellum black, slightly paler than thorax, trapezoidal, 1.4–1.5 times wider than long, microtomentose; apical scutellar setae strong, on small tubercles on dorsal margin of scutellum, lateral scutellar setae much weaker than apical setae but longer and darker than surrounding setae. ***Legs.*** Yellow, hind femur basally and tibia brown; femoral organ present as a very small patch of three sensillae, tibial organ oval, dark, occupying middle half of hind tibia. Wing hyaline, brown tint dorsally from M1; veins brown; ratio of costal sectors C1: C2: C3: C4 – 1 1.7: 1.25: 0.5; haltere yellow. ***Abdomen.*** Paler than thorax, sparsely microtomentose; syntergites 1+2 membranous under scutellum, marginally longer than other tergites. ***Male postabdomen*** (Figs 7, 8). Epandrium large, bulbous, higher than long in lateral view, much wider than high in posterior view, flattened dorsally, with several setae; surstylus 0.6 times as high as epandrium, triangular with a slight anterior curve along length, with broadly rounded apex, surstylus with three or four anterior setae near base and short setae elsewhere; cercus elongate, straight with a narrow ventral projection, extending postero-ventrally, with sparse setae, three setae at tip of cercus longer than others; distiphallus weakly sclerotised.

**Figures 5–9.**
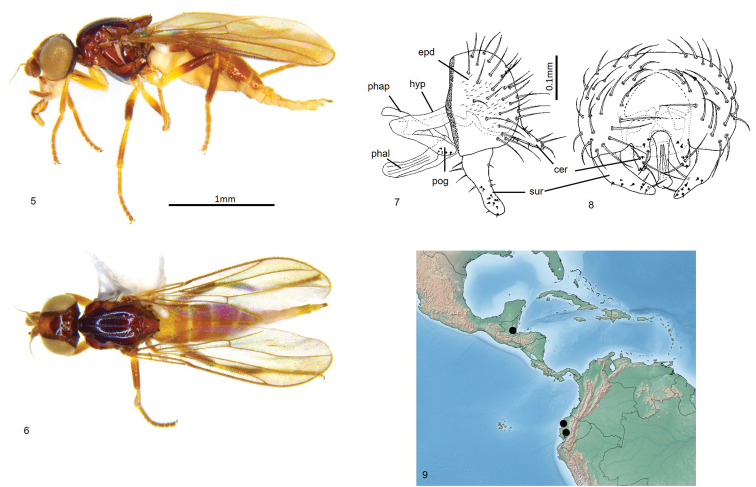
*Enderleiniellacaerulea*. **5** Lateral habitus **6** dorsal habitus **7** male genitalia (lateral) **8** male genitalia (posterior) **9** geographic distribution. Abbreviations: cer – cercus; epd – epandrium; hyp – hypandrium; phal – phallus; phap – phallapodeme; pog – postgonite.

#### Type material.

**Holotype** ♂: BELIZE: Toledo District, Blue Creek (16°12'N, 89°3'W), 23.i.1982, A.T. Finamore, sweeping (LEM). **Paratypes**: same as holotype except 17.i.1982 (1♀, LEM); ECUADOR: Guare Los Rios, vii.1955, Levi-Castillo (1♂, USNM; USNMENT01476001); Manabi Camarones 9.viii.1955, Levi-Castillo (1♂, USNM; USNMENT01476000).

#### Etymology.

The species name is from the Latin *caeruleus* (sky-blue), referring to the type locality.

### Enderleiniella cryptica sp. nov.

Taxon classificationAnimaliaDipteraChloropidae

57576EFC-9A24-50E2-B4A1-D930CFE7EB68

http://zoobank.org/59B9BAAD-E4BE-42F3-9D19-6D80741455DE

Figs 10–14

#### Diagnosis.

Medium Oscinellinae with a shiny frontal triangle and thorax. Mouthparts geniculate. Scutellum with distinct tubercles. Male postabdomen large with parallel sided surstylus.

#### Description.

Total length 2.2–2.5 mm. Overall colour black. ***Head.*** Frontal triangle black, shiny, microtomentose, 0.6–0.65 times length of frons; ocellar tubercle black, shiny, microtomentose; frons brown to black, paler antero-medially; cephalic setae dark, 11–17 fronto-orbital setae well-developed, interfrontal setulae on margin of frontal triangle and fronto-orbital setulae conspicuous, six or seven post-ocellar setulae small; gena yellow, microtomentose, 0.08–0.1 times eye height; face yellow; scape and pedicel yellow, first flagellomere yellow basally and ventrally, darker dorsally and distally, first flagellomere round, arista brown, thin at base, pubescence sparse and short; palpus and clypeus yellow in males; proboscis brown, geniculate. ***Scutum.*** Black, shiny, acrostichal and dorsocentral setae in three punctuate rows, scutum longer than wide; scutellum black, trapezoidal, 1.3–1.5 times wider than long, microtomentose; apical scutellar setae strong, on small tubercles on upper margin of scutellum, lateral scutellar setae much weaker than apical setae but longer and darker than surrounding setae. ***Legs.*** Yellow, hind femur and tibia brown; femoral organ present as row of three or four sensillae, tibial organ oval, dark, occupying middle third of hind tibia. ***Wing.*** Hyaline; veins brown; ratio of costal sectors C1: C2: C3: C4 – 1: 1.7: 1.1: 0.6; haltere yellow. ***Abdomen.*** Paler than thorax, sparsely microtomentose; syntergites 1+2 membranous under scutellum, marginally longer than other tergites. ***Male postabdomen*** (Figs 12, 13). Epandrium large, bulbous, higher than long in lateral view, wider than high in posterior view, with several setae, rounded dorsally; surstylus 0.6 times as high as epandrium, straight, spoon shaped, surstylus with five anterior setae near base and short setae elsewhere; cercus straight with a narrow ventral projection, extending ventrally, cercus with sparse setae, five setae longer than others; distiphallus weakly sclerotised, straight, projecting posteriorly.

**Figures 10–14.**
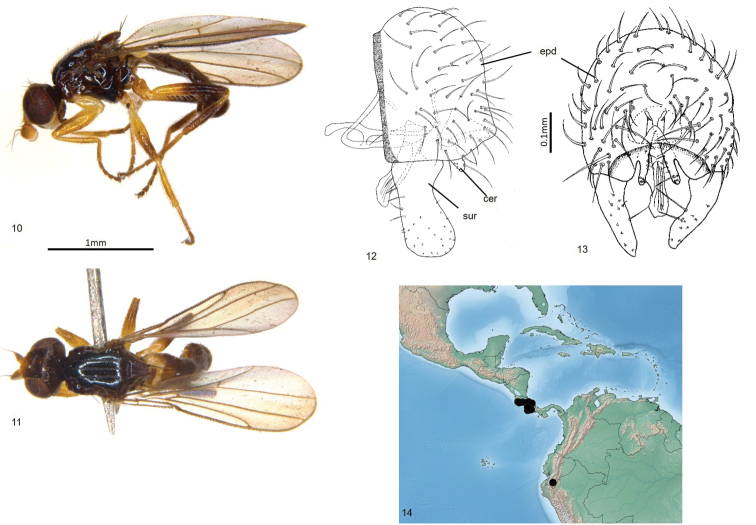
*Enderleiniellacryptica*. **10** Lateral habitus **11** dorsal habitus **12** male genitalia (lateral) **13** male genitalia (posterior) **14** geographic distribution.

#### Molecular data.

Accession numbers MK919190, MK919192, and MK919196.

#### Type material.

**Holotype** ♂: COSTA RICA: Prov Cartago, Cartago, P.N. Barbilla, Camino a Valle Escondido, Rio Dantas, 400–500m, 17.ix.2001, E. Rojas, F. Umaña, Libre, L_N_218000_594300 #64657 (INBio). **Paratypes**: same as holotype (1♂, 1♀, INBio); Higuito, San Mateo, Pablo Schild Coll (1♂, USNM; USNMENT01476002); Prov Alajuela, C.B. Guanacaste-Rincón de la Vieja, Estac. San Gerardo, Send al Perdido, 600m, 16–18.x.2002. D. Briceño, Red. L.N. 317994 384374 #68995 (1♀, INBio); Prov Cartago, P.N. Barbilla, Send. Principal antes de Río Dantas, 200–300m 16.ix.2000 E Rojas, Red de Barrido, L.N. 217400 596700 #58440 (4♂, 2♀, INBio); Prov Cartago, P.N. Barbilla, Send. Principal Río Dantas, 370m, 8.xii.2002, E. Rojas, Red de Golpe, L.N.217250 596250 #70369 (2♂, INBio); Prov Cartago, R.F. Río Pacuara, Turrialba, P.N. Barbilla, Send. Quebrada, 400m 11.x.2001, E. Rojas, Red de Golpe, L.N. 217500 596893 #63565 (1♀, INBio); Prov. Cartago, R.F. Río Pacuare, P.N. Barbilla, Send. Principal a Río Dantas, 370m 22.ix.2001, E. Rojas. W. Arana, R. Madigal, Golpe, L.N. 217500 596893 #64659 (1♀, INBio); Prov Guanacaste, Nandayure, Cerro Azul 1018m, 5.ii.2003, Y. Cardenas. Red de Golpe, L.N. 214769 397000 #7288 (1♀ INBio); Prov. Límon, P.N. Barbilla, Camino a Valle Escondido, Orilla Río Dantas, 400m, 11.x.2001, E. Rojas, Red de Golpe, L.N. 218800 594300 #64954 (1♂, 2♀, INBio); Prov Límon, P.N. Cahuita, Sector Puerto Vargas, Orilla de la playa, Om, 16–17.i.2003, E Rojas, Red de Golpe, L.N.190500 666200 #72723 (1♂, INBio); Prov Límon, P.N. Cahuita, Sector Puerto Vargas, Orilla de la playa, Om, 15.i.2003 E Rojas, Red de Golpe, L.N.190500 666200 #72722 (1♀, INBio); Prov Límon, PN. Barbilla, Sector Casa Negra, 1.5km NO dela Estación, 300m, 13.xii.2002, E. Rojas, Libre, L.N. 219900 598400 #70494 (1♀, INBio); Prov. Punta, Albergue Cerro de Oro, 200m, 4–14.v.1995, E. Alfaro, L.S. 280450 517500 #5919 (11♂, 8♀, INBio); Prov. Punta, Albergue Cerro de Oro, 200m, 5–12.v.1995, M.A. Zumbado, L.S. 279660 518450 #6028 (1♀, INBio); Prov. Punta, Albergue Cerro de Oro, 200m, 5–9.v.1995, B. Gamboa, L.N. 279650 518450 #4745 (1♂, INBio); Prov. Puntarenas, Golfito. P.N. Corcovado, Salida de la Estac. a Río Rincón, 75m 16.x.2002. K. Caballero. Libre, L.S.281050 516800 #71799 (1♀, INBio); Prov Puntarenas, Est. Agujas, Río Agujas,300m, 19–24.iii.1997. A. Azofeifa, L.S. 276750 526550 #46258 (1♀, INBio); Prov. Puntarenas, Est, Río Bonito, 2.3km al O. del Cerro al Gamba, 110m, 17–21.iii.1997. E. Fletes, L.S. 293900 547075 #45597 (1♂, INBio) Prov. Puntarenas R.F. Golfo Dulce, 24km W Piedras Biancas, 200m, xii.1990. P. Hanson (1♂, LEM); Prov Puntarenas, R. Priv. Karen Mogensen. Send. Quebrada Pérez, 315m, 24.ix.2003. W. Porras, Red de Golpe, L.N. 205300 419750 #75433 (9 ♂, 3♀, INBio); Prov Puntarenas, Lepanto, R. Priv. Karen Mogensen. Send. Quebrada Pérez, 315m, 22–23.xi.2003. D. Briceño, Libre, L.N. 205300_419750#74568 (3♂, 2♀, INBio); Prov Puntarenas, Sendero Tres Ríos, 300m, 9.xii.2003. M.A. Zimbado. W. Porras Vega Libre, L.N. 205164 419993 #74577 (7♂, 3♀, INBio); Prov Puntarenas, R. Priv. Karen Mogensen. Send. El Viejo Nisper, 300–500m, 23.xi.2003. Y. Cardenas, Red con Aguamiel, 205600 420300 #74531 (1♀, INBio); ECUADOR: Rio Mulaute, 15km N.E. Sto. Domingo de Colorados, 2.iii.1973, M. & N. Deyrup (1♂, USNM; USNMENT01476003).

#### Etymology.

The species name is from the Latin *crypticus* (hidden), referring to the external similarity of this species to *E.tripunctata*.

### Enderleiniella flavida sp. nov.

Taxon classificationAnimaliaDipteraChloropidae

F7CD226E-9B97-50A4-99CD-CED11CC101EE

http://zoobank.org/41D4A122-BC45-4255-9EA7-F42537546EAD

Figs 3 , 15–19

#### Diagnosis.

Medium Oscinellinae with a shiny frontal triangle and thorax. occiput with single strong bristle behind eye; one anterior and one posterior notopleural bristle Scutellum pale yellow, contrasting in colour with the dark scutum.

#### Description.

Total length 2.2–2.5 mm. Overall colour black. ***Head.*** Frontal triangle black, shiny, 0.6–0.75 times length of frons; ocellar tubercle black, shiny; frons brown to black, paler antero-medially; cephalic setae pale, 7–10 fronto-orbital setae well-developed, interfrontal setulae on margin of frontal triangle and fronto-orbital setulae sparse and small, 4–6 post-ocellar setulae small; gena yellow, microtomentose, 0.08–0.1 times eye height; eye bare; occiput with a strong and stout seta projecting from a short tubercle just dorsal to posterior midpoint of eye; face yellow; scape, pedicel and first flagellomere yellow, first flagellomere round, arista brown, thin at base, pubescence sparse and short; palpus, clypeus and proboscis yellow; proboscis geniculate. ***Scutum.*** Black, shiny, acrostichal and dorsocentral setae in three punctuate rows, notopleural bristle one anterior and one posterior relatively thick and long; outer postalar setae very short, gold, cryptic and fine; dorsocentral setae weak, scutum as long as wide; scutellum yellow, trapezoidal, 1.4–1.6 times wider than long, microtomentose; apical scutellar setae strong, on small tubercles on upper margin of scutellum, lateral scutellar setae as strong as apical setae (Fig. 3). ***Legs.*** Yellow; femoral organ small row of two or three tubercles, tibial organ oval, pale, occupying middle third of hind tibia. ***Wing.*** Hyaline; veins brown; ratio of costal sectors C1: C2: C3: C4 – 1: 2.1: 1.4: 0.6; haltere yellow. ***Abdomen.*** paler than thorax, sparsely microtomentose; syntergites 1+2 membranous under scutellum, marginally longer than other tergites. ***Male postabdomen*** (Figs 17, 18). Epandrium small, higher than long in lateral view, wider than high in posterior view, with several setae; surstylus 0.7 times as high as epandrium, with a slight curve at the base, parallel-sided, apex rounded, surstylus with short setae; cercus broad with three narrow ventral projection, extending ventrally laterally, cercus separated by very narrow anal membrane posteriorly, cercus with sparse setae, one setae longer than others; distiphallus weakly sclerotised.

**Figures 15–19.**
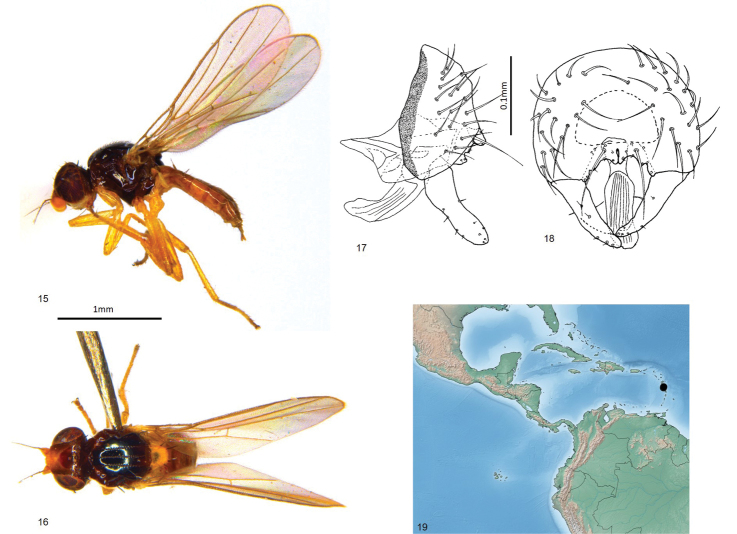
*Enderleiniellaflavida*. **15** Lateral habitus **16** dorsal habitus **17** male genitalia (lateral) **18** male genitalia (posterior) **19** geographic distribution.

#### Type material.

**Holotype** ♂: DOMINICA: S. Chiltern Est, 20.ii.1965, W.W. Wirth (USNM; USNMENT01476004). **Paratypes**: same data as holotype (2♂, 4♀, USNM; USNMENT1476005-USNMENT01476009); DOMINICA: W.I. 2mi E. Ponte Casse, 5.x.1966, R.J. Gagne. Bredin-Archibol-SmithsonianBio.Surv.Dominica (1♀, USNM; USNMENT01476010); DOMINICA: W.I. d’LeauGommier, 16.iii.1965, W.W. Wirth (1♂, USNM; USNMENT01476011) DOMINICA: St. David: Emerald Pool, rainforest, 20.xi.1994, L. Masner (2♀, LEM).

#### Etymology.

The species name is from the Latin *flavida* (yellow), referring to the colour of the scutellum.

#### Remarks.

It is the first time that a strong and stout seta projecting from a short tubercle just dorsal to posterior midpoint of eye on occiput has been described in the Chloropidae. It could be that this seta has the same evolutionary origin as those found on many other Chloropidae species that possess a long (but not stout) seta on the postgena.

### Enderleiniella longiventris

Taxon classificationAnimaliaDipteraChloropidae

(Enderlein, 1912)

E2E8959E-EE2A-5352-9CCC-3B56A0866FC5

Figs 1 , 2 , 20–22 , 23–27

Tricimba longiventris Enderlein, 1911: 207 (type locality: Costa Rica).
Enderleiniella longiventris : Becker 1912: 192.

#### Description.

Total length 2.5–3.0 mm. Overall colour black. ***Head*** Frontal triangle black, shiny, microtomentose, 0.55–0.6 times length of frons; ocellar tubercle black, shiny, microtomentose; frons brown to black, paler antero-medially; cephalic setae dark, 12–15 fronto-orbital setae well-developed, interfrontal setulae on margin of frontal triangle and, seven or eight post-ocellar setulae small, posterior setulae proclinate; gena yellow, microtomentose, 0.08–0.09 times eye height; face yellow; scape and pedicel yellow, first flagellomere yellow apico-dorsally darker, first flagellomere round, arista brown, thin at base, pubescence sparse and short; palpus yellow clypeus and proboscis brown, proboscis geniculate and thin (Fig. 1). ***Scutum.*** Black, pruinose, acrostichal, and dorsocentral setae in three punctuate rows, scutum longer than wide; outer postalar bristle very robust and long, scutellum black, trapezoidal, 1.2 times wider than long, microtomentose, rugose; apical scutellar setae strong, on tubercles on upper margin of scutellum, lateral scutellar setae much weaker than apical setae, on tubercles, longer than surrounding setae (Fig. 2). ***Legs.*** Yellow, hind femur and hind tibia brown; femoral organ arranged in one line of three strong sensillae, tibial organ oval, pale, occupying middle third of hind tibia. ***Wing.*** Hyaline; veins brown; ratio of costal sectors C1: C2: C3: C4 – 1: 1.3: 1: 0.4; haltere yellow. ***Abdomen.*** As dark as thorax, sparsely microtomentose; syntergites 1+2 membranous under scutellum, slightly longer than other tergites. ***Male postabdomen*** (Figs 23–27). Remnant of sternite 6 present; Epandrium large, bulbous, higher than long in lateral view, wider than high in posterior view, with several setae; surstylus half the height of epandrium, straight, parallel-sided, blunt-ended, surstylus with one or two anterior setae near base and short setae elsewhere; cercus elongate, straight with a narrow ventral projection, extending ventrally, cercus with sparse setae, one or two setae longer than others; distiphallus weakly sclerotised, straight, blunt ended.

**Figures 20–22.**
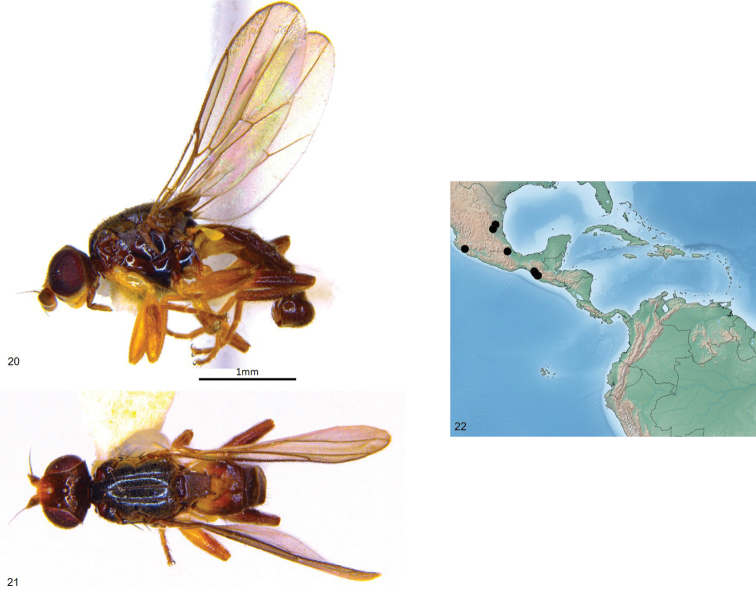
*Enderleiniellalongiventris*. **20** Lateral habitus **21** dorsal habitus **22** geographic distribution.

**Figures 23–27.**
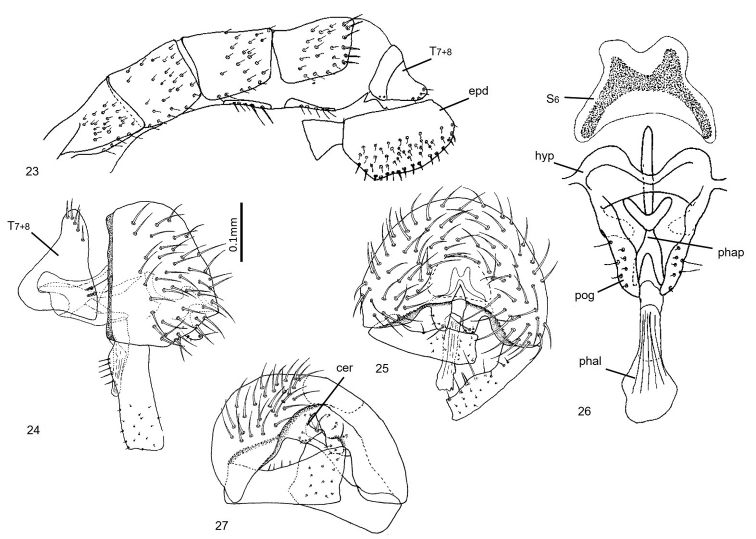
*Enderleiniellalongiventris*. **23** Male abdomen (lateral) **24** male genitalia (lateral) **25** male genitalia (posterior) **26** male genitalia (ventral) **27** male genitalia (posteroventral). Abbreviations: cer – cercus; hyp – hypandrium; phal – phallus; phap – phallapodeme; pog – postgonite; S_6_ – remnant sternite 6; T_7+8_ – tergite 7+8.

#### Molecular data.

Accession number MK919191.

#### Type material.

**Holotype** ♂: COSTA RICA, H. Schmidt (Warsaw).

#### Other material examined.

COSTA RICA: Cartago: Rio Grande de Orosi nr Tapanti Natl. Pk. 1100–1150m, floodplain and forest, 9.x.1999, S. Marshall & M. Buck. (1♂, DEBU; DEBU00103967). MEXICO: Chiapas: 7 km N Cacahoatan, 22.iv.1983, W.N. Mathis (2♂, USNM; USNMENT01476013-USNMENT01476014, USNMENT01476026); Chiapas: Finca Prusia, 33 km S Jaltenango, 1000m, 10–12.v.1985, W.N. Mathis (3♂, 1♀, USNM; USNMENT01476015-USNMENT01476018); Chiapas: 9 km S Union Juaréz, 23.iv,1983, W.N. Mathis (1♂, USNM; USNMENT01476019); Chiapas, 20–25 mi. N Huixtla 3000’, 4.vi.1969, B.V. Peterson (6♂, 1♀, CNC); Colima: 7mi NE Colima, 3.xii.1948 (1♀, USNM; USNMENT01476020); Tamaulipas: Liera, 10–6-1956, reared ex *Colocasia* sp. probably *antiquorom* (1♂, 2♀, USNM; USNMENT01476021-USNMENT01476023); San Luis Potosi: Naranjo, xii.1960, A. Fabergé, reared ex aroid flower (1♀, USNM; USNMENT01476024); Veracruz: Fortin de las Flores, 952m, 02.v.1985, W.N. Mathis (1♂, USNM; USNMENT01476025).

#### Remarks.

Although the holotype was collected in Costa Rica, most examined specimens of *E.longiventris* for this study are from Mexico. This is one of two species of *Enderleiniella* (the other is *E.maculata*) whose known range extends into the Nearctic Region, with specimens recorded in the Mexican states of San Luis Potosi and Tamaulipas.

### Enderleiniella maculata sp. nov.

Taxon classificationAnimaliaDipteraChloropidae

34C83030-6142-58A6-AF31-9D39E55A3B28

http://zoobank.org/CE01F49F-CDF5-43A7-91D2-83D89AE7BC08

Figs 4 , 28–30 , 31–34

#### Diagnosis.

Medium Oscinellinae with a pruinose frontal triangle and thorax. Wing with a dark apical spot. Male postabdomen small, not excessively higher than pre-epandrium.

#### Description.

Total length 2.4–2.9 mm. Overall colour black. ***Head.*** Frontal triangle black, pruinose, 0.5 times length of frons; ocellar tubercle black, microtomentose; frons yellow, heavily microtomentose; cephalic setae pale, eleven fronto-orbital setae weak-developed, interfrontal setulae on margin of frontal triangle, eight post-ocellar setulae small, posterior ocellar setae proclinate; gena white anteriorly, black posteriorly, microtomentose, 0.08 times eye height; eye hairy; face yellow; scape, pedicel brown, first flagellomere brown, yellow basoventrally, arista black, thin at base, pubescence sparse and short; palpus yellow in male, brown in female, proboscis and clypeus brown.

***Scutum.*** Black, pollinose, acrostichal, and dorsocentral lines pruinosity with setae in 3 faint punctuate rows, notopleural bristle one anterior and two posterior relatively thick; outer postalar setae strong, black; dorsocentral setae strong, scutum 1.2 times longer than wide; scutellum brown, trapezoidal,1.35–1.50 times wider than long, microtomentose, rugose; apical scutellar setae strong, on small tubercles on upper margin of scutellum, lateral scutellar setae small, barely longer than other dorsal setae. ***Legs.*** Yellow, mid femur, hind femur and hind tibia black; femoral organ small patch on baso-anterior part of mid femur, tibial organ linear, pale, occupying middle quarter of hind tibia. ***Wing.*** Hyaline with distinct apical brown spot (Fig. 4); veins brown; ratio of costal sectors C1: C2: C3: C4 – 1: 1.7: 1.1: 0.6; haltere white. ***Abdomen.*** Paler than thorax, sparsely microtomentose; Abdominal syntergites 1+2 slightly longer than other tergites. ***Male postabdomen*** (Figs 31–34). Epandrium small, higher than long in lateral view, wider than high in posterior view, with many setae; surstylus 0.7 height of epandrium, straight, parallel-sided; cercus narrow with long ventral projection, cercus with sparse setae; Distiphallus weakly sclerotised.

**Figures 28–30.**
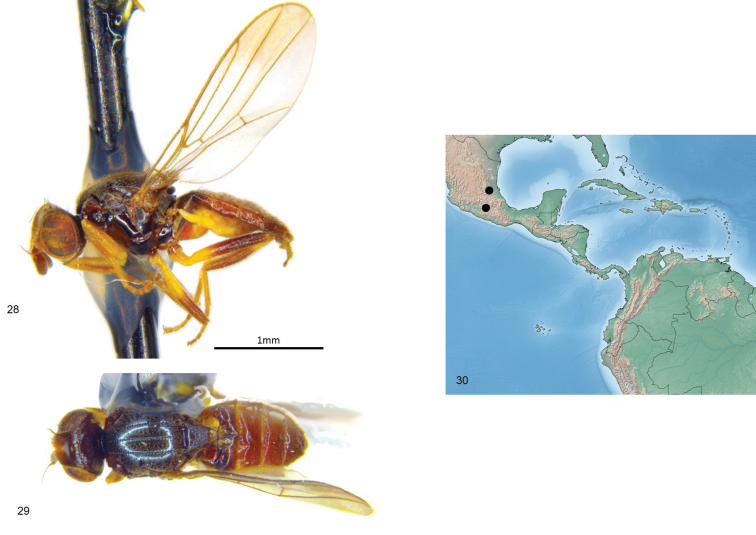
*Enderleiniellamaculata*. **28** Lateral habitus **29** dorsal habitus **30** geographic distribution.

**Figures 31–34.**
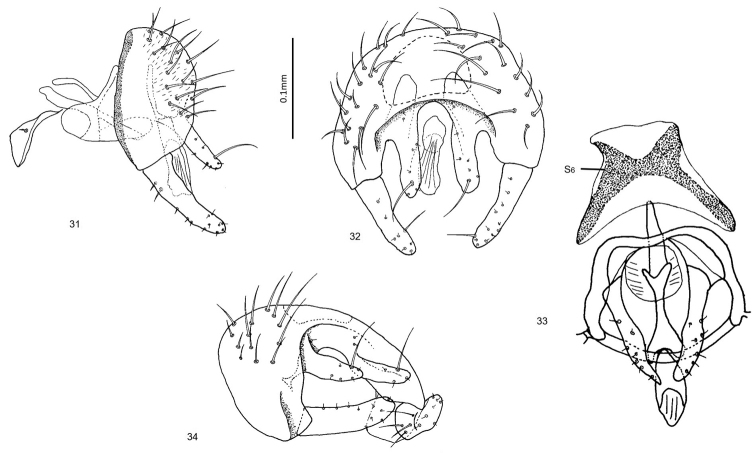
*Enderleiniellamaculata*. **31** Male genitalia (lateral) **32** male genitalia (posterior) **33** male genitalia (ventral) **34** male genitalia (posteroventral).

#### Type material.

**Holotype** ♂: MEXICO: San Luis Potosi: Xilitla, 1800’, 24.vii.1954, J.G. Chillcott (CNC); Paratypes: same data as holotype (5♂, 7♀, CNC); Guerrero, Taxco, 8mi NE, 5154’, 8.viii.1954, J.G. Chillcott (1♀, CNC).

#### Etymology.

The species name is from the Latin *maculatus* (spotted), referring to the wing pattern.

### Enderleiniella marshalli sp. nov.

Taxon classificationAnimaliaDipteraChloropidae

9868E800-38F7-57EE-9D13-C6B7013134D0

http://zoobank.org/6DBF3CF0-A1A4-49C1-B11B-0C9D38853E0F

Figs 35–37 , 38–41

#### Diagnosis.

Small Oscinellinae with a shiny frontal triangle and sparsely pruinose thorax. Scutellum trapezoidal. Male postabdomen small with parallel sided surstylus.

#### Description.

Total length 2.0–2.5 mm. Overall colour black. ***Head.*** Frontal triangle black, shiny, microtomentose, 0.7–0.8 times length of frons; ocellar tubercle black, shiny, microtomentose; frons black, paler antero-medially; cephalic setae dark, 6–8 fronto-orbital setae weak-developed, interfrontal setulae on margin of frontal triangle and fronto-orbital setulae sparse and small, five or six post-ocellar setulae small; gena white, margin of gena black, microtomentose, 0.08–0.1 times eye height; eye hairy; face yellow; scape yellow, pedicel yellow to brown, first flagellomere black, reniform, arista yellow distally darkening brown, thin at base, pubescence sparse and short; palpus, clypeus and proboscis brown to black in females, palpus yellow, clypeus and proboscis brown to black in male; proboscis regular. ***Scutum.*** Black, pruinose, acrostichal and dorsocentral setae in three punctuate rows, notopleural bristle one anterior and one posterior relatively thick and long; outer postalar setae strong, black; dorsocentral setae strong, scutum as long as wide or marginally longer than wide; scutellum black, trapezoidal, 1.4–1.5 times wider than long, microtomentose, rugose; apical scutellar setae strong, on small tubercles on upper margin of scutellum, lateral scutellar setae as small, marginally larger than other dorsal setae. ***Legs.*** Yellow, fore-tarsi, mid and hind femur and tibia dark distally; femoral organ present as a row of three sensillae, tibial organ linear, brown, paler than leg, occupying middle quarter of hind tibia. ***Wing.*** Hyaline; veins brown; ratio of costal sectors C1: C2: C3: C4 – 1: 1.6: 1.2: 0.6; haltere, white. ***Abdomen.*** paler than thorax, sparsely microtomentose; syntergites 1+2 membranous under scutellum, marginally longer than other tergites. ***Male postabdomen*** (Figs 38–40). Epandrium small, higher than long in lateral view, wider than high in posterior view, with many setae; surstylus 0.7 height of epandrium, with slight curve basally, parallel-sided, with short setae elsewhere; cercus small, extending ventrally, with sparse setae, one seta longer than others; hypandrium open; distiphallus weakly sclerotised.

**Figures 35–37.**
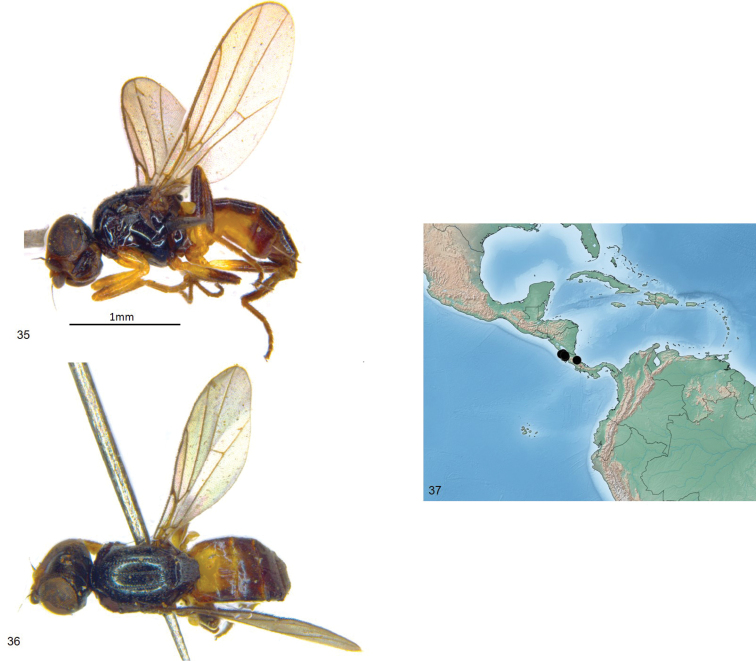
*Enderleiniellamarshalli*. **35** Lateral habitus **36** dorsal habitus **37** geographic distribution.

**Figures 38–41.**
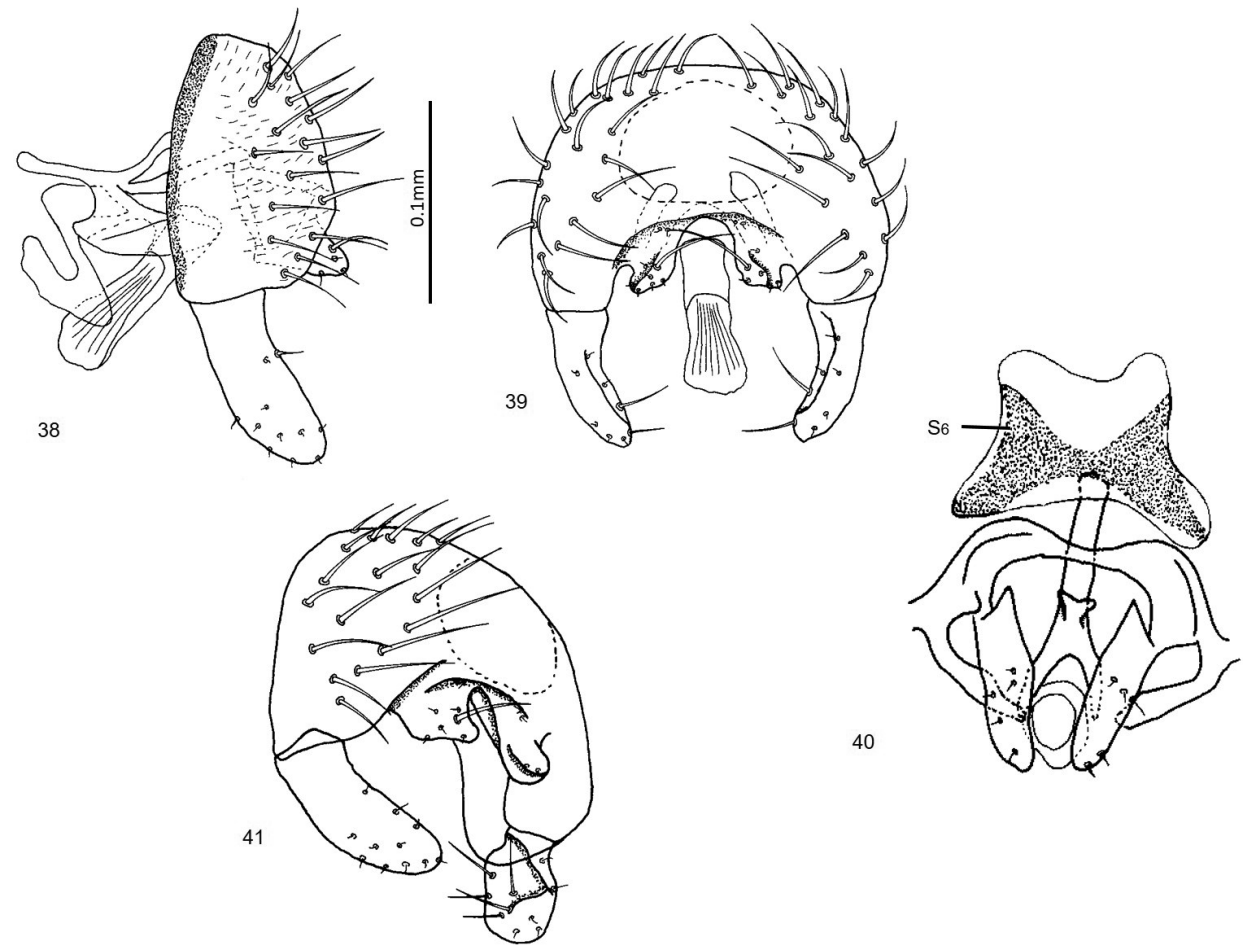
*Enderleiniellamarshalli*. **38** Male genitalia (lateral) **39** male genitalia (posterior) **40** male genitalia (ventral) **41** male genitalia (posteroventral).

#### Type material.

**Holotype** ♂: COSTA RICA: Guanacaste: Cacao Field Stn., carrion traps, 700m, 13–15.ii.1996, S. A. Marshall (DEBU). **Paratypes**: same data as holotype (4♀, DEBU); Guanacaste: Estacion Santa Rosa, 300m,16.ii.1996, S. Marshall, Borde del Rio, L_N_313000_359800 #6920, INBIO CRI002239665 (1♀, INBio). ♂: COSTA RICA: Prov Guanacaste, Cañas, Palmira, Sector Rio Corobici. 224m, 10–15.x.2002, J.D. Gutiérrez, Libre, L_N_281200_416500 #71957 (INBio). Paratypes: Prov Guanacaste, Bagaces, Fortuna, Z.P. Miravalles, Send. Cabro Muco, 980m, 1–15.viii.2002. J.D. Gutiérrez, Red de Golpe L_N_299151_410000 #64536 (1♂, INBio); same data as except 12.iv.2002, L_N_299151_410000 #67730 (1♀, INBio); Prov Cartago, Cartago, P.N. Barbilla, Camino a Valle Escondido, Rio Dantes, 400–500m, 17.ix.2001, F. Rojas, f. Umaña, Libre, L_N_281200_594300 #64657 (1♀, INBio).

#### Etymology.

This species is named in honour of Steve Marshall, collector of the type series, in recognition of his contributions to our knowledge of Central American acalyptrate Diptera.

### Enderleiniella maya sp. nov.

Taxon classificationAnimaliaDipteraChloropidae

CB1CC70C-4B82-5405-ADCB-317919186242

http://zoobank.org/74FBCE7B-165B-4BF5-BFA8-AA98D3018169

Figs 42–46

#### Diagnosis.

Small Oscinellinae with a shiny frontal triangle and thorax. Scutellum rectangular with distinct apical scutellar bristles. Male postabdomen large with parallel sided surstylus with broadly rounded apex.

#### Description.

Total length 2.4–2.6 mm. Overall colour black. ***Head.*** Frontal triangle black, shiny, microtomentose, 0.7 times length of frons; ocellar tubercle black, shiny, microtomentose; frons brown to black; cephalic setae dark, eight or nine fronto-orbital setae well-developed, interfrontal setulae on margin of frontal triangle and fronto-orbital setulae sparse, five orbital setulae small, posterior orbital setae proclinate, proclinate bristle on vertex between postocellar and inner vertical; gena yellow, microtomentose, 0.09–0.1 times eye height; face yellow; scape and pedicel yellow, first flagellomere yellow, black dorsally and distally, first flagellomere round, arista brown, thin at base, pubescence sparse and short; palpus yellow, proboscis and clypeus brown, proboscis geniculate. ***Scutum.*** Black, pruinose, acrostichal and dorsocentral setae in three punctuate rows, scutum longer than wide; scutellum black, rectangular, 1–1.1 times wider than long, microtomentose, rugose; apical scutellar setae strong, on tubercles 0.2 times as long as length of scutellum, on upper margin of scutellum, lateral scutellar setae much weaker than apical setae but longer and darker than surrounding setae, on small tubercles. ***Legs.*** Yellow, mid and hind femurs basally and tibia brown; femoral organ present as a small patch of three sensillae, tibial organ linear, dark, occupying middle third of hind tibia. ***Wing.*** Hyaline; veins brown; ratio of costal sectors C1: C2: C3: C4 – 1: 1.5: 1: 0.5; haltere yellow. ***Abdomen.*** Same colour as abdomen, sparsely microtomentose; syntergites 1+2 membranous under scutellum, marginally as long as tergites 3 and 4 together. ***Male postabdomen*** (Figs 44, 45). Epandrium large, bulbous, higher than long in lateral view, wider than high in posterior view, with several setae; surstylus 0.6 times height of epandrium, parallel-sided straight with broadly rounded apex, surstylus with short setae; cercus elongate, straight with a narrow ventral projection, extending postero-ventrally cercus with sparse setae, one seta longer than others; distiphallus weakly sclerotised.

**Figures 42–46.**
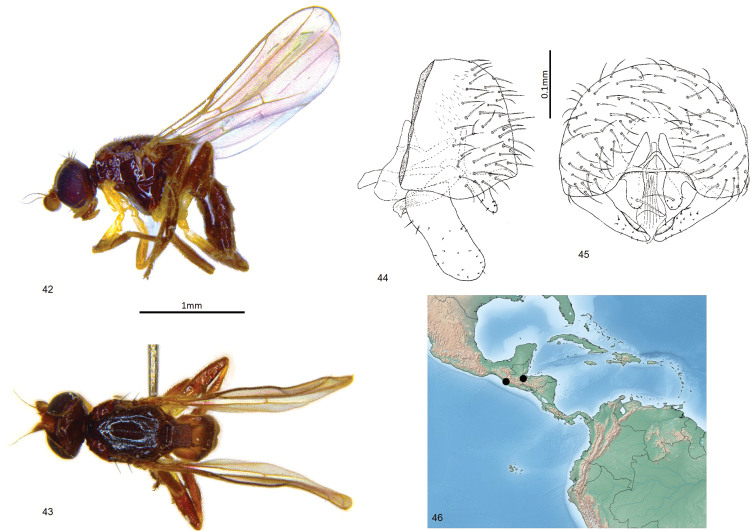
*Enderleiniellamaya*. **42** Lateral habitus **43** dorsal habitus **44** male genitalia (lateral) **45** male genitalia (posterior) **46** geographic distribution.

#### Type material.

**Holotype** ♂: GUATEMALA: Departamento Izabal: Las Escobas, 15.vii.1986, L. Lesage (CNC). **Paratypes**: Same as holotype (1♂), MEXICO: Chiapas, Rio Izapa, 21.iv.1983, W.N. Mathis (1♀, USNM; USNMENT01476012).

#### Etymology.

The species name, to be treated as a noun in apposition, refers to the Maya people whose culture has been dominant in this region for more than 1000 years.

### Enderleiniella punctata sp. nov.

Taxon classificationAnimaliaDipteraChloropidae

776EC718-330D-571E-8167-09BA47796A64

http://zoobank.org/57607AE9-E81F-4342-9825-2F346703C2BA

Figs 47–52

#### Diagnosis.

Small Oscinellinae with a shiny frontal triangle and thorax. Scutellum trapezoidal with very small apical tubercles bristles. Tergite 3 with medial setulae arising from large distinct punctate sockets. Male postabdomen small with parallel sided surstylus.

#### Description.

Total length 2.1 mm. Overall colour black. ***Head.*** Frontal triangle black, pollinose, shiny posteriorly, 0.6 times length of frons; ocellar tubercle black, microtomentose; occiput black, shiny; frons brown, yellow antero-medially, heavily microtomentose; cephalic setae dark, eight fronto-orbital setae weak-developed, interfrontal setulae on margin of frontal triangle, six post-ocellar setulae small; gena white, margin of gena black, microtomentose, 0.1 times eye height; eye hairy; face yellow; scape pedicel yellow, first flagellomere yellow, black anterodorsally, large, quadrate, arista yellow basally darkening distally, thin at base, pubescence sparse and short; palpus and clypeus yellow, proboscis brown, geniculate. ***Scutum.*** Black, shiny, acrostichal and dorsocentral setae in 3 faint punctuate rows, notopleural bristle one anterior and two posterior relatively thick; outer postalar setae strong, black; dorsocentral setae strong, scutum 1.1 times longer than wide; scutellum black, trapezoidal, 1.7 times wider than long, microtomentose, smooth; apical scutellar setae strong, on small tubercles on upper margin of scutellum, lateral scutellar setae as large, twice as long as other dorsal setae. ***Legs.*** Yellow, all tarsi, mid and hind femur and tibia dark distally; femoral organ present as a small patch of three sensillae, tibial organ linear, brown, paler than leg, occupying middle quarter of hind tibia. ***Wing.*** Hyaline; veins brown; ratio of costal sectors C1: C2: C3: C4 – 1: 1.2: 1.4: 0.6; haltere white. ***Abdomen.*** Paler than thorax, sparsely microtomentose; Abdominal syntergites 1+2 elongate, tergite 3 with medial setulae arising from large distinct punctate sockets (Fig. 49). ***Male postabdomen*** (Figs 49–51). Epandrium small, higher than long in lateral view, wider than high in posterior view, with many setae; surstylus 0.7 height of epandrium, straight, parallel-sided, surstylus with one anterior setae near base and short setae elsewhere; cercus elongate with narrow, long ventral projection, cercus with sparse setae, one seta longer than others; hypandrium open; distiphallus weakly sclerotised, straight, blunt ended.

**Figures 47–52.**
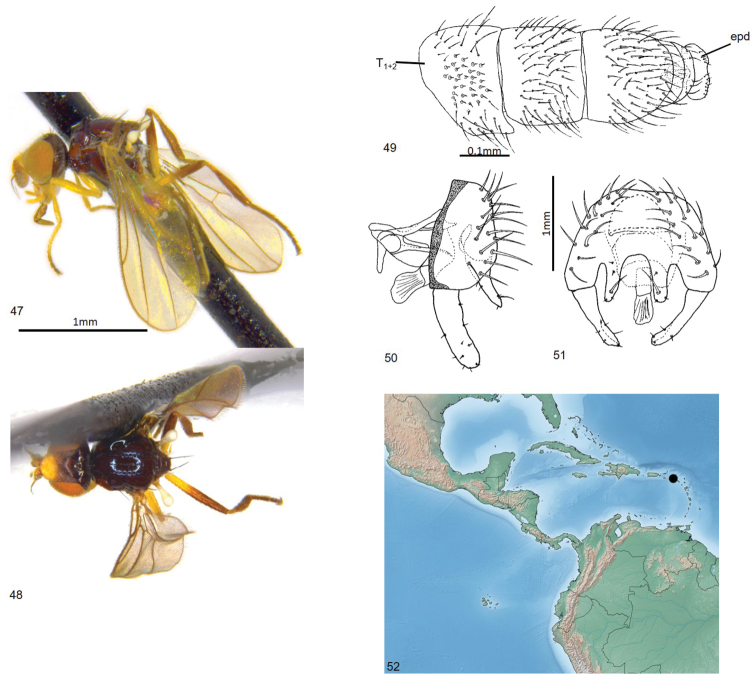
*Enderleiniellapunctata*. **47** Lateral habitus **48** dorsal habitus **49** male abdomen (dorsal) **50** male genitalia (lateral) **51** male genitalia (posterior) **52** geographic distribution. Abbreviations: Epd – epandrium; T_1+2_ – tergites 1+2.

#### Type material.

**Holotype** ♂: BOLIVIA: Santa Cruz: Andres lbanez, Potrerillo (17°40'S, 63°27'W), 438m, yellow pan trap B-17, 13–16.v.1997, L. Masner (CNC).

#### Etymology.

The species name is from the Latin *punctatus* (punctured), referring to the structure of the third abdominal tergite.

### Enderleiniella tripunctata

Taxon classificationAnimaliaDipteraChloropidae

(Becker, 1916)

33BCF99D-CC26-545D-9648-B656B44EAF35

Figs 53–57

Anoscinella tripunctata Becker, 1916: 448 (type locality: Costa Rica: Higuito, San Mateo).
Enderleiniella tripunctata : Duda 1930: 77.

#### Description.

Total length 1.9–2.6 mm. Overall colour black. ***Head.*** Frontal triangle black, shiny, microtomentose, 0.6–0.7 times length of frons; ocellar tubercle black, shiny, microtomentose; frons brown to black, paler medially; cephalic setae dark, eight or nine fronto-orbital setae well-developed, interfrontal setulae on margin of frontal triangle and fronto-orbital setulae sparse, 5–7 orbital setulae small, posterior setae proclinate; gena yellow, microtomentose, 0.09–0.1 times eye height; face yellow; scape and pedicel yellow, first flagellomere yellow basally and ventrally, black dorsally and distally, first flagellomere quadrate, arista brown, thin at base, pubescence sparse and short; palpus yellow in male, brown in female, proboscis and clypeus brown. ***Scutum.*** Black, shiny, acrostichal and dorsocentral setae in three punctuate rows, scutum longer than wide; scutellum black, trapezoidal, 1.2–1.3 times wider than long, microtomentose; apical scutellar setae strong, on small tubercles on upper margin of scutellum, lateral scutellar setae much weaker than apical setae but longer and darker than surrounding setae. ***Legs.*** Yellow, mid and hind femora basally and tibia brown; femoral organ present as a small patch of four sensillae, tibial organ linear, dark, occupying middle quarter of hind tibia. ***Wing.*** Hyaline, brown tint dorsally from M1+2; veins brown; ratio of costal sectors C1: C2: C3: C4 – 1: 1.9: 1.4: 0.5; haltere yellow. ***Abdomen.*** Slightly paler than abdomen, sparsely microtomentose; syntergites 1+2 membranous under scutellum, marginally longer than other tergites. ***Male postabdomen*** (Figs 55, 56). Epandrium large, bulbous, higher than long in lateral view, wider than high in posterior view, with several setae; surstylus half the height of epandrium, clavate with a slight anterior curve along length, with broadly rounded apex, surstylus with three or four anterior setae near base and short setae elsewhere; cercus elongate, straight with a narrow ventral projection, extending postero-ventrally cercus with sparse setae, three or four setae longer than others; distiphallus weakly sclerotised, straight, blunt ended.

**Figures 53–57.**
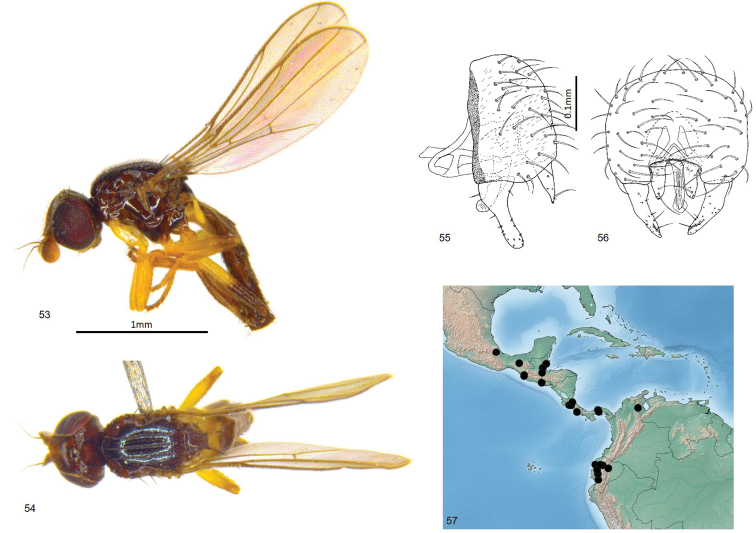
*Enderleiniellatripunctata*. **53** Lateral habitus **54** dorsal habitus **55** male genitalia (lateral) **56** male genitalia (posterior) **57** geographic distribution.

#### Molecular data.

Accession number MK919194

#### Type material.

**Holotype** ♂: COSTA RICA: [Provincia de San José, about 9°56'N, 84°32'W, 200m asl], Higuito: San Mateo, 1914, P. Schild (Budapest).

#### Other material examined.

BELIZE: BARC, near San Pedro Colombia (16°17'N, 88°58'W), Malaise trap and yellow pans, 10–12.iii.2002, J. Skevington (1♂, LEM); Stan Creek District: Silk Grass Creek (16°54'N, 88°26'W), 3.iv.1993, W.N. Mathis (1♂, USNM; USNMENT01476027); Toledo District: Blue Creek (16°12'N, 89°3'W), sweeping, 17.i.1982, A.T. Finnamore (1♀, LEM); COSTA RICA: Puntarenas: 24 km W Piedras Blancas (8°47'N, 83°15'W), 200m, Malaise trap, xi.1990, P. Hanson (1♀, USNM; USNMENT01476028); Puntarenas: 3 km SW Rincon (9°55'N, 84°13'W), 10m, Malaise trap, x–xii.1990, P. Hanson (1♂, USNM; USNMENT01476029); Higuito: San Mateo, P. Schild (1♂, 2♀, USNM; USNMENT01476030-USNMENT01476032); Heredia: 3 km S Puerto Viejo OTS-La Selva, 100m, Malaise traps, xi.1992, P. Hanson (1♀, LEM); ECUADOR: Manabi, La Palma, viii.1955, Levi-Castillo (1♀, USNM); Manabi, Camarones, 9.ix.1955, Levi-Castillo (1♂, USNM; USNMENT01476033); Rio Mulaute, 15 km NE Santo Domingo de los Colorados, 2.iii.1973, M. & N. Deyrup (1♂, 3♀, USNM; USNMENT01476034-USNMENT01476036, USNMENT01476041); Domingo de los Colorados, 5.iii.1973, M. & N. Deyrup (1♂, USNM, USNMENT01476066); Napo: Napo-Pastaza, Levi-Castillo (1♀, USNM; USNMENT01476037); Balao Guayas, xii.1955, Levi-Castillo (3♀, USNM; USNMENT01476038-USNMENT01476040); Guayas: Naranjal, xii.1955, Levi-Castillo (1♀, USNM; USNMENT01476042); Guare: Los Rios,viii.1955. Levi-Castillo (1♀, USNM; USNMENT01476043); Guayas: Taura, xii.1955, Levi-Castillo (3♀, USNM; USNMENT01476044-USNMENT01476046); Pichincha Manabi, viii.1955, Levi-Castillo (3♀, USNM; USNMENT01476047-USNMENT01476049); EL SALVADOR: San Salvador, x.1965,N.L.H. Krauss (1♀, USNM; USNMENT01476050); GUATEMALA: Departamento Izabal: Las Escobas, 15.vii.1986, L. LeSage (1♂, CNC); MEXICO: Chiapas: 7 km N Cacahoatan, 22.iv.1983, W.N. Mathis (1♂, 1♀, USNM; USNMENT01476051-USNMENT01476052); Chiapas: Rio Izapa, 21.iv.1983, W.N. Mathis (1♀, USNM; USNMENT01476055); Chiapas: Puenta Macalapa, light trap, 22.v.1964, F.S. Blanton (1♀, USNM; USNMENT01476056); Vera Cruz: Cordoba, vii.1965, N.L.H. Krauss (1♀, USNM; USNMENT01476057); PANAMA: Gamboa: Pipeline Road, Malaise traps, vii.1967, W.W. Wirth (1♂, 1♀, USNM; USNMENT01476058-USNMENT01476059); Gamboa: Rio Agua Salud, vii.1967, W.W. Wirth (1♀, USNM; USNMENT01476060); Summit, ix.1946, N.H.L. Krauss (1♀, USNM; USNMENT01476061); Tabogal, 26.ii.1912, A. Busck (1♀, USNM; USNMENT01476062); PERU: Canet, 17.v.1941, P.A. Berry (2♀, USNM; USNMENT01476063-USNMENT01476064); VENEZUELA: Zulia: El Tucuco, 45 km SW Machiques, 5–6.vi.1976, A.S. Menke & D. Vincent (1♀, USNM; USNMENT01476065).

### Enderleiniella tumescens sp. nov.

Taxon classificationAnimaliaDipteraChloropidae

5617E285-CF13-5C39-9AA1-070500A9EF2F

http://zoobank.org/E8291025-CEAB-4EA9-A6D9-586CE6869F89

Figs 58–63

#### Diagnosis.

Small Oscinellinae with a shiny frontal triangle and sparsely pruinose thorax. Occiput with dorsolateral pubescent swelling just behind outer vertical bristle. Scutellum trapezoidal with very small apical tubercles bristles. Male postabdomen small with parallel sided surstylus.

#### Description.

Total length 2.3–2.5 mm. Overall colour black. ***Head.*** Frontal triangle black, pruinose, 0.7 times length of frons; ocellar tubercle black, microtomentose; occiput with distinct pubescent swelling (Fig. 33); frons brown, yellow antero-medially, heavily microtomentose; cephalic setae dark, eleven fronto-orbital setae weak-developed, interfrontal setulae on margin of frontal triangle, six post-ocellar setulae small; gena white, microtomentose, 0.09 times eye height; eye hairy; face yellow; scape, pedicel yellow, first flagellomere and arista missing in both type specimens; palpus yellow, proboscis and clypeus brown. ***Scutum.*** Black, shiny, acrostichal and dorsocentral lines pruinose with setae in three faint punctuate rows, notopleural bristle one anterior and two posterior relatively thick; intra-alar setae strong, black; dorsocentral setae strong, scutum 1.2 times longer than wide; scutellum brown, trapezoidal, 1.7 times wider than long, microtomentose, smooth; apical scutellar setae strong, on small tubercles on upper margin of scutellum, lateral scutellar setae as large, twice as long as other dorsal setae. ***Legs.*** Yellow, hind tibia darker; femoral organ present as a small patch of three sensillae, tibial organ oval, pale, occupying middle third of hind tibia. ***Wing.*** Hyaline; veins brown; ratio of costal sectors C1: C2: C3: C4 – 1: 1.9: 1.2: 0.5; haltere white. ***Abdomen.*** Paler than thorax, sparsely microtomentose; abdominal syntergites 1+2 elongate. ***Male postabdomen*** (Figs 61, 62). Epandrium small, higher than long in lateral view, wider than high in posterior view, with many setae; surstylus 0.8 height of epandrium, straight, parallel-sided; cercus elongate, narrow with long ventral projection, cercus with sparse setae, one seta near apex long; hypandrium open; distiphallus weakly sclerotised, blunt-ended.

**Figures 58–63.**
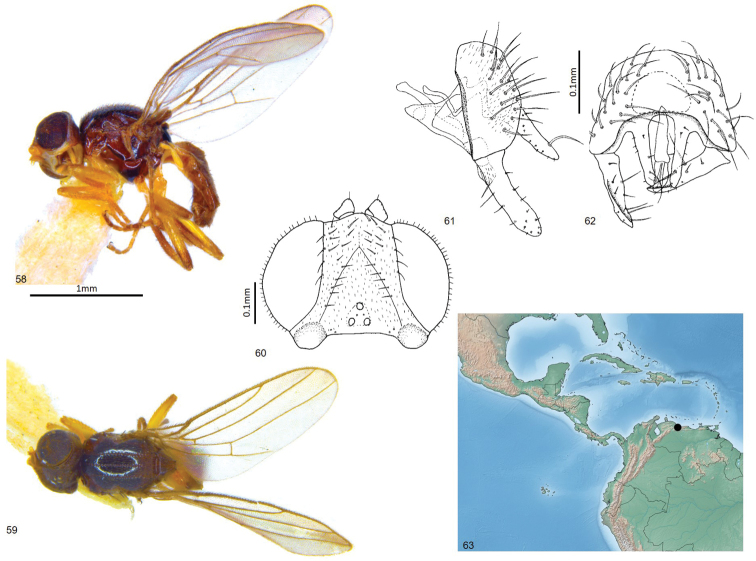
*Enderleiniellatumescens*. **58** Lateral habitus **59** dorsal habitus **60** head (dorsal) **61** male genitalia (lateral) **62** male genitalia (posterior) **63** geographic distribution.

Female unknown.

#### Molecular data.

Accession number MK919195

#### Type material.

**Holotype** ♂: VENEZUELA: San Esteban, xii.1939, P. J. Anduze (USNM; USNMENT01476067). **Paratype**: same data as holotype (1♂, USNM; USNMENT01476068).

#### Etymology.

The species name is from the Latin *tumescens* (swollen), referring to the distinctive structure of the occiput.

### Enderleiniella wheeleri sp. nov.

Taxon classificationAnimaliaDipteraChloropidae

63687580-1864-5B80-A225-14A1E3C998E7

http://zoobank.org/059F617B-9B40-43C1-B7B7-33E61C86E6F3

Figs 64–68

#### Diagnosis.

Small Oscinellinae with a shiny frontal triangle and thorax. Mouthparts geniculate. Scutellum trapezoidal with very small apical tubercles bristles. Male postabdomen small with a triangular surstylus.

#### Description.

Total length 1.9–2.0 mm. Overall colour black. ***Head.*** Frontal triangle black, pruinose, 0.7 times length of frons; ocellar tubercle black, microtomentose; frons brown, yellow antero-medially, microtomentose; cephalic setae dark, six or seven fronto-orbital setae weak-developed, interfrontal setulae on margin of frontal triangle, six or seven ocellar setulae small, posterior setae proclinate; gena white, microtomentose, 0.07–0.08 times eye height; eye hairy; face yellow; scape, pedicel, first flagellomere brown to black, first flagellomere subquadrate, arista black, thin at base, pubescence sparse; palpus yellow, clypeus and proboscis black in males. ***Scutum.*** Black, shiny, acrostichal and dorsocentral setae in three faint punctuate rows, notopleural bristle one anterior and two posterior relatively thick; outer postalar setae strong, black; dorsocentral setae strong, scutum 1.2 times longer than wide; scutellum black, trapezoidal, 1.6 times wider than long, microtomentose, smooth; apical scutellar setae strong, on very small tubercles on dorsal margin of scutellum, lateral scutellar setae as large, twice as long as other dorsal setae. ***Legs.*** Yellow, mid and hind femur and tibia dark distally; femoral organ a line of three sensillae, tibial organ linear, brown, paler than leg, occupying middle third of hind tibia. ***Wing.*** Hyaline; veins brown; ratio of costal sectors C1: C2: C3: C4 – 1: 1.7: 1.2: 0.6; haltere white. ***Abdomen.*** Paler than thorax, sparsely microtomentose; abdominal syntergites 1+2 slightly longer than other tergites. ***Male postabdomen*** (Figs 66, 67). Epandrium higher than long in lateral view, wider than high in posterior view, with many setae; surstylus 0.5 height of epandrium, triangular, with a slight posterior curve, surstylus with one anterior seta near base and short setae elsewhere; cercus narrow with long ventral projection, cercus with sparse setae, one seta longer than others; distiphallus weakly sclerotised, straight, blunt-ended.

**Figures 64–68.**
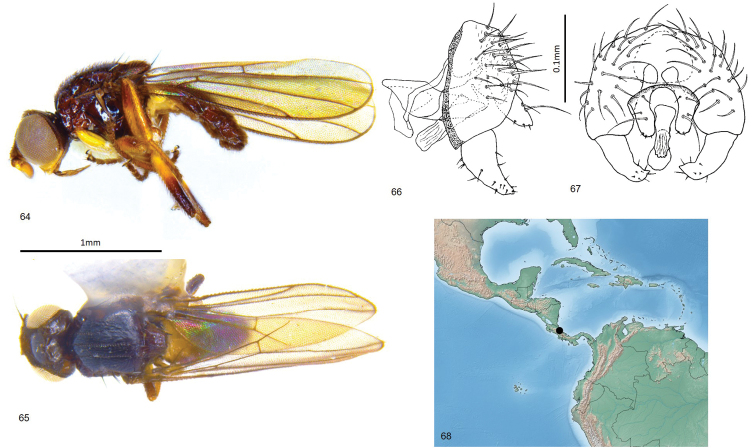
*Enderleiniellawheeleri*. **64** Lateral habitus **65** dorsal habitus **66** male genitalia (lateral) **67** male genitalia (posterior) **68** geographic distribution.

Female unknown.

#### Type material.

**Holotype** ♂: COSTA RICA: Prov. Cartago, Turrialba, P.N. Barbilla, 2km S. de Est. por la Quebrada, 200–300m, 25.ix.2000, E. Rojas, Red Barrido, L.N. 217500 596893 #58442 (INBio; INBIO0003466073). **Paratype**: same as holotype (1♂, INBio; INBIO0003466202).

#### Etymology.

This species is named in honour of Terry Wheeler, in recognition of his contributions to our knowledge of New World Chloropidae.

## Discussion

*Enderleiniella* was distinguished by previous authors (Becker 1912, Duda 1930) on the basis of the incised lines on the scutum, the absence of the alula, and the reduced anal angle of the wing. The scutal character is consistent, although variable in known species, where the incised lines are much clearer in certain species such as *E.longiventris* but much more subtle in in species like *E.tumescens*. The alula is present, but small, in most species of *Enderleiniella* and the anal angle varies but it is not as pronounced as in many oscinelline genera (Fig. 4). As with many oscinelline genera, the phylogenetic relationships of *Enderleiniella* within the subfamily are unclear. The incised lines on the scutum are shared with the species-rich and cosmopolitan genus *Tricimba* Lioy, but the well-developed pronotal carina because of the rounded shape of the back of the head and the distinct postpronotal sulcus of *Enderleiniella* are absent in species of Neotropical and Nearctic *Tricimba* that have been examined. There are also some male genitalic differences between the two genera such as the overall size of the epandrium compared with the abdomen and the presence of the remnant of sternite 6 in several species. These pronotal and postpronotal characters are shared with other Neotropical oscinelline genera and may be indicative of close relationship but it would be premature to speculate without a broader and more comprehensive phylogenetic analysis. Some characters (linear gena, anteromedial-posterolateral placement of vertical setae, stout, tuberculate scutellar setae, large male epandrium) are shared with the Neotropical genus *Agrophaspidium* Wheeler & Mlynarek, and the two may be related (Wheeler and Mlynarek 2008). *Enderleiniellaflavida* has 1+1 notopleural setae, as in *Agrophaspidium*, and the structure of the scutellum in that species is intermediate between the two genera. It would be difficult to construct a cladogram of species-level relationships within *Enderleiniella* based on morphological characters because most of the known species are defined on autapomorphies, with few synapomorphic character states uniting species within the genus.

**Figure 69.**
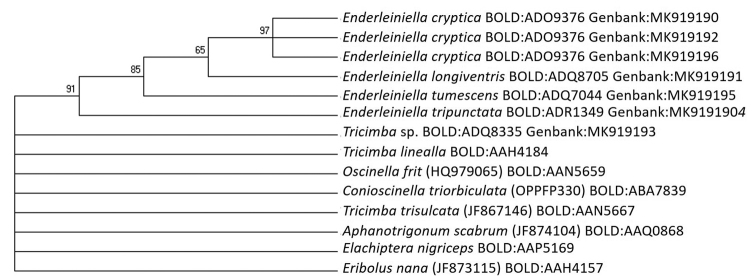
Maximum likelihood tree based on Cytochrome oxidase I barcode fragment using GTR+G+I evolutionary model. Bootstrap values above the branches are based on 1000 replicates of the analysis. Branches with less than 50% support are collapsed. Accession numbers in parentheses.

The molecular barcode data also supports *Enderleiniella* as a valid genus distinct from *Tricimba*. All the sequences from *Enderleiniella* cluster together in 91% of the bootstrap replicates whereas fewer than 50% of the bootstrap replicates supported *Tricimba* as sister to *Enderleiniella.* If the clades with < 50% support are collapsed (Fig. 69), there remains only support to maintain *Enderleiniella* with completely unresolved relationships with all the other outgroup species. I must emphasise that this should not be considered a true phylogenetic analysis of *Enderleiniella*. *Enderleiniella* is a valid genus based on morphological and molecular (COI barcode fragment) support. This revision also demonstrates the need for revision and redefinition of the limits of *Tricimba* and Chloropidae using an integrated taxonomic approach.

Four of the eleven described species of *Enderleiniella* are known from only one or two specimens and mostly from single localities in an area extending from Mexico to Bolivia. This suggests that additional sampling effort in the Neotropical Region will result in discovery of more undescribed species.

The biology of *Enderleiniella* was unknown prior to this study. Even now, there is limited ecological information on the species assigned to the genus. Four specimens of *E.longiventris* were reared from plants of *Colocasia* (Araceae) and flowers of an unidentified aroid plant in Mexico, although there is no indication as to whether the larvae were phytophagous in live plants or secondary invaders of dead or damaged plant tissues. Other specimens examined in this study were collected in a broad range of localities and habitats, from primary rainforest to disturbed dry areas.

## Supplementary Material

XML Treatment for Enderleiniella

XML Treatment for Enderleiniella caerulea

XML Treatment for Enderleiniella cryptica

XML Treatment for Enderleiniella flavida

XML Treatment for Enderleiniella longiventris

XML Treatment for Enderleiniella maculata

XML Treatment for Enderleiniella marshalli

XML Treatment for Enderleiniella maya

XML Treatment for Enderleiniella punctata

XML Treatment for Enderleiniella tripunctata

XML Treatment for Enderleiniella tumescens

XML Treatment for Enderleiniella wheeleri
